# The implementation of pharmacogenomics into UK general practice: a qualitative study exploring barriers, challenges and opportunities

**DOI:** 10.1007/s12687-020-00468-2

**Published:** 2020-05-28

**Authors:** I. Rafi, I. Crinson, M. Dawes, D. Rafi, M. Pirmohamed, F. M. Walter

**Affiliations:** 1 St George’s, University of London, London, UK; 2 Department of Family Practice, University of British Columbia, Vancouver, Canada; 3 University of Birmingham, Birmingham, UK; 4 Molecular and Clinical Pharmacology, University of Liverpool, Liverpool, UK; 5 The Primary Care Unit, Department of Public Health & Primary Care, University of Cambridge, Cambridge, UK

## Abstract

Pharmacogenomics describes interpatient genetic variability in drug responses. Information based on whole genome sequencing will soon open up the field of pharmacogenomics and facilitate the use of genomic information relating to drug metabolism and drug responses. We undertook a qualitative study, aiming to explore the potential barriers, opportunities and challenges facing the implementation of pharmacogenomics into primary care. Semi-structured interviews were undertaken with 18 clinical participants (16 GPs and 2 other clinicians). All interviews were recorded and transcribed verbatim. Using a thematic analysis approach, data items were coded, ordered and themes constructed. Most participants were aged 55–60 years and worked as part-time clinical GPs with other clearly defined roles. The emerging themes covered several areas of concern, including the following: the utility of pharmacogenomics and the value of introducing such testing into primary care; how to educate the primary care workforce and ‘mainstream’ pharmacogenomics; the ethical, legal and social aspects of pharmacogenomics and its impact on patients; and potential impacts on the healthcare system particularly around economics and informatics. Most participants had concerns about pharmacogenomics and felt that there were a number of barriers and challenges to its implementation into routine primary care. Most striking were their concerns around the cost-effectiveness of using pharmacogenomics in primary care. At the same time most recognised the increasing availability of direct-to-consumer testing, and felt that this would drive the need to understand the ethical and social implications of using genomic information in primary care. This study has raised important issues that need to be considered when planning the implementation of pharmacogenomics into clinical practice. Prior to the implementation of genomic testing into day-to-day practice in UK primary care, it is important that considerations around education, cost-effectiveness and informatics are addressed, as well as the impact on patients.

## Background

The 100,000 Genome project is a translational research project (Genomics England n.d.) which is stimulating NHS clinicians, researchers and policymakers to consider how genomic medicine will be mainstreamed across all specialties, including General Practice. Health Education England (HEE) is addressing this concern by evaluating the genomics educational needs of General Practitioners (GPs) through surveys and feedbacks (HEE Genomics Programme: Engaging Primary Care n.d.). The use of genomic information at the time of drug prescribing is a potential application, but one that is not currently implemented in any systematic way in UK clinical practice. ‘Pharmacogenomics’ describes the effect of the genome on drug response. There is acceptance that while the future implementation of prescribing based on the use of genomic data is likely, we will need to consider key issues such as clinical effectiveness and cost-effectiveness (Hillman and Dale 2017; Hayward et al. 2017; Walter et al. 2014) before there is widespread adoption and clinical confidence in General Practice.

### Why is the study of pharmacogenomics clinically useful?

Whole-genome sequencing (WGS) and other more targeted forms of genotyping are beginning to enable testing for relevant specific genetic variants that influence drug-prescribing choices; these, in turn, can be personalised to the individual. This is important for both drug efficacy and drug safety.

The risk of an adverse event should be used to aid choice of both drug and dosage (Ventola 2013) and potentially reduce toxicity. This is important since the clinical and economic cost to the NHS is high when 6–7% of hospital admissions are due to adverse drug reactions (Adverse Drug reactions- summary n.d.). The PRACTICE Study reported that 30% and 47% of patients receiving 5 or more and 10 or more medications, respectively, had prescribing or monitoring errors in a 12-month study period (Avery et al. 2013).

Drug dose and response can be determined by an individual’s drug metabolizing capacity (Adverse Drug reactions- summary n.d.; Professor Bill Newman 2017). Enzyme systems such as cytochrome p450 provide examples of this variability, as they influence commonly prescribed drugs such as tamoxifen (CYP2D6), codeine (CYP2D6) and clopidrogel (CYP2C19) (Chang et al. 2015). Research looking at rates of hospitalisation in a primary care population showed that patients who were ultra-rapid CYP2D6 metabolisers had an increased rate of hospitalisation compared with CYP2D6 extensive metabolisers (Takahashi et al. 2017). Another key example is anticoagulation, as randomised controlled trials have shown that genotype-guided warfarin dosing is superior to standard dosing with respect to the time in the therapeutic INR range (Pirmohamed et al. 2013; Dahal et al. 2015). Further work in UK primary care examining this issue is ongoing (Kendrick et al. 2017).

GPs are not likely to be aware of The Clinical Pharmacogenetics Implementation Consortium (The Clinical Pharmacogenetics Implementation Consortium n.d.) (CPIC), which is working to produce guidelines that use genomic data that may lead to actionable prescribing decisions for specific drugs. This is an initiative that uses the Pharmacogenomics Knowledge Base resource (Pharmacogenomics Knowledge Base resource 2017) around the impact of genetic variation on drug response.

GP views around the use of genomic information and its value to clinical care are therefore important to understand (Bouwman et al. 2009; Christensen et al. 2016), in order to deliver effective implementation of pharmacogenomics approaches into routine clinical practice in primary care. The aim of this hypothesis-generating qualitative study was to identify potential barriers, challenges and opportunities to the implementation of pharmacogenomics into UK General Practice.

## Materials and methods

### Participant recruitment

Recruitment was undertaken using a convenience sample, with most participants known professionally to the researcher (IR) either as clinical, academic or RCGP colleagues. Most of the participants were approached either face-to-face or by correspondence. A study information sheet was provided to introduce the work.

### Data collection

All participants were verbally consented, and afterwards provided with a written consent form. All agreed to audio-recording of the interview (see “Appendix” for the interview guide). The interviews were conducted by telephone between April and June 2017 at the interviewee’s convenience. Each interview lasted up to 60 min and was guided by a short topic guide, developed specifically for this study and informed by a literature review. Each transcript was transcribed by an author (IR or DR), and then sent to the participant for any feedback or clarification; only two participants opted not to receive and comment on their transcript. Explicitly personal identifiable information were not included in the data analysis or study reports.

### Analytical approach

Data were subjected to a thematic analytic approach (Ritchie et al. 2003). The transcripts were read multiple times by the first author (IR), with any early themes colour-highlighted. During the analysis process, codes were attached to sections of excerpts of interview transcripts, inter-related, and then linked ideas emerging from the transcripts were combined together as sub-themes. These sub-themes were subsequently grouped into a more encompassing draft thematic framework. Following an iterative review carried out by another author (FW), the final framework was agreed upon.

## Results

### Participants

A total of 18 people were interviewed, with the majority being GPs (*n* = 16). Although the GPs’ modal age group was 50–59 years, efforts were made to recruit early career GPs to interview a spectrum of participants by age and therefore length of service. Most of the GPs worked as part-time clinicians, often combining this with other professional roles such as academic or policy work; one was a recently retired GP. A scientific curator (age 30–39, 12 years as a scientist) and a public health medicine researcher (age 50–59 years, 20 years as a public health researcher) were also interviewed.

### Thematic framework

Seven broad themes emerged from the analysis and are detailed as follows:

1. *Pharmacogenomics: could it be useful in general practice?*

The key issues to emerge were a lack of knowledge and awareness and concerns around the evidence-base and utility of the data that might be used. *‘I’ve not heard the term (Pharmacogenomics), it’s something new to me’ (R15, Locum GP),* and *‘I think those terms do make sense but even I don’t know the difference between genetics and genomics, so I wouldn’t know what pharmacogenetics is as opposed to pharmacogenomics’ (R11, academic GP). ‘I think one of the challenges is about what level of evidence do we need to justify using these tests in routine practice…. If you look at the randomised controlled trials, they provide a degree of justification in terms of informing those, but not so much in terms of reducing clinical adverse effects’ (R13, academic GP).*

These data suggest that GPs may have little knowledge around pharmacogenomics, and there is therefore a general need for information about what contribution genomics could offer to improve the safety and effectiveness of prescribing in routine clinical primary care.

2. *How will patient factors influence implementation?*

Many interviewees made interesting observations around patient factors influencing pharmacogenomics, including the effects of personalised medicine and its impact on patient perspectives and shared decision-making. They valued patient-centred care but were anxious around extra workload pressures following implementation of personalised medicine.

Genetics was seen as one of several elements contributing to ‘personalising medicine’, *‘personalised medicine is about interpreting all the things in your context, the genetic element is one element, and it’s not the overriding element’ (R4, informatics expert).* The application of pharmacogenomics in personalised medicine was apparent to many. *‘I think GPs would be wise to understand, or at least be familiar with, the new role of genetic information in personalising medication’ (R2, RCGP clinical lead).*

Patient anxiety and workload pressures were also important considerations. *‘So yes, it’ll definitely create more work I think both for us and for secondary care as well, and probably increase patient anxiety if they come wanting to know the significance of information’ (R15 Locum GP)*. Any interviewee mentioned, *‘there’s something a bit ethical about it as well in terms of exactly what genomic data you are getting and whether there’s any data that predicts future risk that you may or may not want to have known’ (R17, Clinical Lead).*

Presentation of the evidence to patients was considered important for a meaningful discussion, and the importance of shared decision making was raised by some, *’you have still got to develop a shared understanding of it between you; you have still got to have the same process of shared decision making’ (R10, GP Academic),* and *‘I think it’s something about the general area around informed choice and rational decision’ (R5, Public Health Researcher).*

3. *How important is pharmacogenomics education?*

Many felt that the level of pharmacogenomics knowledge that a primary care healthcare professional might need would vary depending on the level of complexity around clinical management they were responsible for, *‘we are talking about different responses to drugs which will include a more detailed understanding of the genetic issues’ (R2, GP with informatics knowledge).* Professional differences and needs were also highlighted. *‘Practice GPs will seek education about it; ...another driver which is really crucial is the RCGP curriculum’ (R9, GPSI genetics).*

Patients knowledge and beliefs were considered by many, *‘My patient cohorts are quite well informed – they educate me a lot of the time’ (R16, Clinical Fellow)*. Others mentioned the traditional approach of an expert opinion as the best source of information, *‘always really grateful when someone came in to talk to us about say BRCA risk or something like that – it filled the gap’ (R16,Clinical Fellow).* Some people felt that *‘understanding of genetics is very variable, and people have all sorts of belief about the terms genetics and what genes and gene tests can tell them.’ (R9 GPSI genetics).*

Some felt that involving other health care professionals such as pharmacists would be important, and it would require uniform dissemination of genomics education and competency needs. ‘*The other aspect is to take it out of the hands of GPs, and delegate to nurse practitioners or pharmacists, they would do it, [so] wider MDT sharing of role, this might be something that a pharmacist do it in a practice [especially] if doing large numbers at a time’ (R12, a RCGP medical director).*

Some focused on the vital role of education for successful implementation*:* indeed, education was often considered to go beyond the types of programmes aimed at either patients or professionals. *‘I mean from an educational programme, [would] have to put more through schools, universities, degrees you know, medical schools’ (R1,GP Principal).*

Responses here highlighted the importance of skill-mix and delegation of roles which could underpin a strategic approach to the delivery of pharmacogenomics education.

4. *What are the barriers to mainstreaming pharmacogenomics in clinical practice?*

The key challenge for the NHS was universally considered to be how the use of genomic information could be ‘mainstreamed’ (i.e. as part of normal practice) into general practice. It was clear that respondents thought that there would need to be a pool of GPs who could offer advice. However, the barriers to implementation included the fact that in General Practice at present, only small numbers of GPs understand the concepts. ‘*It will be rare to find a GP who is up to speed with pharmacogenomics... genomics whatever’ (R6, Academic GP)*.

Many felt that there was a need for both individual training and the ‘mainstreaming’ process facilitated through generating an expert pool in general practice: *‘[it would] make sense to have a collaboration of Primary Care Physicians interested around pharmacogenomics‘ (R3, RCGP lead*) *or that ‘every GP would need some sort of training. Mainly brand new GPs would have enough knowledge but I suspect not – maybe in 5 years’ time when it’s more in forefront but not yet’ (R1,GP Principal). ‘But I think at the moment most GPs would need lots of training to use it’ (R1,Clinical Fellow)* and inherent scepticism *‘..[just] think about trying to translate some of the apparent promise of the approach into practice’ (R10, Academic GP).*

Despite the obvious barriers, there was some acceptance that this was just likely to happen: *‘this is new and yes, it will become mainstream, and it will become part of part of our clinical decision-making like everything else, because it’s going to be’ (R7, Digital lead)*. ‘*It could revolutionise our practice, it’s hugely exciting for the future and GPs, rather than saying this is too much for us, we could be at the forefront of this’ (R12, a RCGP medical director).*

It was interesting that GPs perceived pharmacogenomics tests being used as tool as part of day-to-day clinical practice with the potential for General Practice to lead the way.

5. *Social (and family) implications of pharmacogenomics information*

Many GPs were concerned about insurance loading for relatively minor conditions, and that genomic data may be used to make such decisions around insurance premiums that could have potential repercussions for family members.

Genomic information was generally considered to be different to other forms of clinical information. For example, ‘insurance loading’, i.e. paying an extra premium based on personal medical data is something that worries both patients and GPs on behalf of their patients, and was frequently discussed: ‘*obviously it has implications for life insurance’ (R16, Clinical fellow).* Of importance was how many GPs anticipated insurance loading for what seemed relatively minor conditions.

The familial nature of genomic data was also frequently highlighted: *‘I suppose the genetic component that needs explaining is the repercussions for the family, any sort of genetic information can’t be seen in isolation from the family’ (R6,GP academic).* The tension between ownership of personal and family information and ‘societal’ ownership seemed problematic: ‘*So I would want to put that in, it’s owned by society*’ *(R4, informatics expert),* and: ‘*there is social disparity in requesting of (genetic) testing and accessing the testing and that somehow that knowledge could have an effect on family relationships. I think the issues around genomic data is broader, and I think that’s around the impact it has on other people and someone’s future health*’ *(R2, Clinical Lead).*

Reflections like these highlight the perceived familial importance of genomics and the considerations to be taken into account around sharing.

6. *Cost-effectiveness*

With current workload pressures in UK general practice, it was clear that incorporating genomics in practice with the potential benefits was welcome provided the implementation considered cost-effectiveness as a priority.

Issues such as commissioning decisions based on cost-effectiveness were considered to be really important. The balance between financial costs, cost-effectiveness and opportunity costs was mentioned by many participants: ‘*If all these things add up into patient benefits more than other interventions of a similar opportunity costs and similar financial costs, then good. But I wouldn’t want to [adopt it] just because it’s a wonder of biotechnology*’ *(R11, GP academic), and:* ‘*I think for primary care the key issues are going to be clinical utility and cost effectiveness. There are some barriers in terms of commissioning at the moment, and the way commissioning is siloed is going to be one of the main barriers and those are one of the main question marks over pharmacogenomics before it’s mainstream*’ *(R9, GPSI genetics).* However, the current working environment was not considered ideal: *‘CCGs at the moment would be hugely concerned by the workload… it’s a major workforce crisis across the country and struggling to provide enough appointments to see people at the moment, and the thought of having extra work for GPs….*’ *(R1, a RCGP medical director).*

Many felt that approaches to implementation built on established models would be crucial. One felt that the UK could learn about implementation from countries with differing healthcare systems. *‘The Dutch Pharmacogenomics working group have published guidelines for the clinician based on genotype’ (R8, Scientist).* Many interviewees discussed the value of testing in primary care, including examples of application, improving efficacy whilst reducing toxicity and confidence in the use of such approaches. There was some discussion about how pharmacogenomics could offer value, and it was generally deemed to be dependent on the available expertise: *‘I think it will be more important for things like anticoagulants, side effects, cancer therapies, less so for antihypertensive and statins*’ *(R5, Public Health Researcher)*.

There was general agreement about the potential of using a pharmacogenomics approach*: ‘to be able to prescribe medicines with a confidence that [it brings]‘ (R4, informatics expert),* and *‘To have a better outcome, target people more effectively’ (R1, GP Principal)*. ‘*Anything that would help efficacy, it would definitely have the potential to be taken up by GPs.’ (R9, GPSI genetics’.* This point was important and relevant to the discussion about supporting implementation into practice.

7. *Informatics*

There was agreement about the importance of electronic capture of genomic information, anxiety around a new coding system and concern around the principles of data sharing.

Interviewees discussed the application of informatics to pharmacogenomics. In particular, the sensitivity of personal genomics information recorded on a patient’s primary care electronic health record: ‘*it’s the record that is kept by the patient, follows the patient unlike hospital systems*’ *(R1, GP principal).* Many felt that limitations around expertise in coding could be an issue: ‘*I would be worried about would be the coding within the system to make sure we are correctly capturing the right information*’ (R1, GP Principal), with concerns that the information being recorded could be highly sensitive: ‘*concerns about data security is higher with genetic data because it has implications on a criminal type of basis not just for the data subject*’ *(R4, informatics expert).* There was frustration that the recording of family history has not been a success, and some questioned whether genomic information be recorded more successfully. ‘*The reality is we’re talking about genomic information [yet] we haven’t even got decent family history data so how are we going to use genomics if we can’t even incorporate family history?*’ *(R14, Locum GP).* Furthermore, new coding systems due for implementation do not appear to incorporate plans for recording or coding. ‘*SNOMED CT codes, the one that are supposed to be integrated in primary care [soon], they are absolutely not adequate in recording genomic information. They are very patchy, [and] relate to only certain codes*’ *(R9, GPSI genetics).*

‘*it is potentially a problem I think... if the implications of the genomics test affects family members, because we are very good at recording in one patient’s notes but actually because of confidentiality etc. it’s very difficult to ensure all the right people have cross referencing for that result.*’ *(R12, a RCGP Medical Director).* This point reiterates the issue was around data sharing.

## Discussion

### Main findings

This interview study investigating the implementation of pharmacogenomics into UK General Practice has highlighted a number of barriers and challenges. Although some participants recognised the potential opportunities for pharmacogenomics, they all recognised the implementation challenges such as harnessing the *utility* of pharmacogenomics and the *value* of introducing such testing into primary care. Other issues that were considered were around how to *educate* the primary care workforce and *‘mainstream’* pharmacogenomics. Also of importance were the *ethical, legal and social aspects* of pharmacogenomics and its *impact on patients*, and potential impacts on the healthcare system particularly around *cost-effectiveness* and *informatics.*

### Comparison with existing literature

These findings from interviews with UK clinicians correlate with findings from other countries, for example with Canadian primary care providers’ perceptions around personalised genomic medicine (Carroll et al. 2016). The themes raised may be of particular use to policy makers and commissioners. In order to translate and implement findings from pharmacogenomics research into clinical practice, a multi-pronged approach will be needed (Bartlett et al. 2014). Ideally, this should be facilitated by tailored education for primary care clinicians, and the development of electronic decision support systems to enable the integration of genomic and clinical information into the clinical healthcare records (Julia et al. 2010). Beyond the NHS, there are other drivers to clinician and patient ‘mainstreaming’ of pharmacogenomics including commercial companies and the private sector. Direct to consumer genomic (DTC) testing is now widely available, and our findings confirm that there is commercial variation around how such genomic data are handled or utilised, not only in the UK but also from an international perspective(Baroncini et al. 2016; Rafi et al. 2009; Bernhardt et al. 2012; Bartlett et al. 2012). Patients are increasingly using these approaches, and their reasons, such as having a family history of a disease, may enable them to feel empowered by holding this type of pharmacogenomics information (van der Wouden et al. 2016).

Our findings demonstrated that appropriate genomics educational needs of primary care clinicians should be provided, to facilitate understanding of the utility and value of a novel approach to patient management, such as pharmacogenomics, before implementation can be successful. While the Gen-Equip (Paneque et al. 2017) modules of education provide a good resource for European GPs, our findings suggest that information needs to be timely, easily available and succinct. A multi-faceted approach has been promoted as a means of engagement for both GPs (Houwink et al. 2015; Lopes-Junior et al. 2017) and other clinicians including pharmacists and secondary care clinicians (Johansen Taber and Dikinson 2014).

‘Mainstreaming’ pharmacogenomics into the NHS is likely to require expert clinical support. As clinical genetics are unlikely to have the capacity to manage demand (Blashki et al. 2014), secondary care physicians (for example, clinical pharmacologists) will need to perform pharmacogenomics testing and support primary care. This has been simulated in the USA which improved physician’s confidence in prescribing decisions (Overby et al. 2015). However, the lack of clinical knowledge when adding genomic information to US primary care workload has been evaluated in a pilot randomised trial and indicated that WGS may prompt the primary care provider to initiate clinical actions of unclear value. For example, if screening was initiated, or detailed advice given, to change healthcare behaviour for a perceived increase risk in cardiovascular disease or diabetes, when that risk was not above population risk (Vassy et al. 2017).

The findings also demonstrated a range of views around the ethical, legal and social factors surrounding pharmacogenomics. While some felt that pharmacogenomic information was no different to other healthcare data with regard to confidentiality and sharing with external bodies, others felt that such data were sensitive personal data which need to be managed carefully with the potential for discrimination, particularly around insurance (Wauters and Van Hoyweghen 2016). These issues are going to be particularly pertinent in the setting of the General Data Protection Regulation (EU) 2016/679. In North Carolina, USA, insurers have recently declined cover for WGS (although pharmacogenomics testing could be done without WGS), as its utility was considered low. There was also concern about the impact of pharmacogenomics on shared decision-making, similar to the findings from a recent US study which demonstrated the importance of purpose, context and deliberation when incorporating genomic risk assessments into population screening programs (Nicholls et al. 2016). This study suggested that members of a target patient population could engage meaningfully about their acceptability and utility of genomic information (Nicholls et al. 2016). In another study, there was limited discussion on how different populations (ethnic differences) might react to their metaboliser status which might lead to exclusion of drug prescribing for that individual (Kaphingst et al. 2015).

Our study findings demonstrated the known implementation challenges for all genetic and genomics advances (Wauters and Van Hoyweghen 2016), namely the need for an evidence base (including the relative importance of ethnicity in pharmacogenomics clinical decision making), more experience with testing, facilitated integration into the electronic medical record and enhanced knowledge and understanding by clinicians as well as by patients. Real-life examples of where pharmacogenomics has been implemented with pre-emptive genotyping in the USA include the Vanderbilt and St. Jude Children’s Research Hospital and will provide the basis to overcoming these implementation challenges in the UK NHS (Manolio et al. 2013).

### Strengths and limitations of the study

This was one of the first studies to explore GP views on pharmacogenomics in the UK. The participants were predominantly GPs working in the UK and included a spectrum of GPs at different stages of their careers. Using a qualitative approach, with individual interviews, enabled an in-depth understanding and elucidation of the barriers and facilitators to successful implementation of pharmacogenomics in UK primary care practice. Recruitment of more participants, both GPs and other clinicians such as practice nurses and pharmacists, might further our understanding. These early findings from a small participant group could be further explored and confirmed with a larger survey, aiming to recruit a more representative group of primary care clinicians.

### Implications for practice

Clinical decision support may represent the optimal model to utilise genomic information; this applies to a general practice population both in the UK and internationally. The barriers to implementation ranged from lack of knowledge, to issues relating to storage and access of information, to clinical utility underpinned by an evidence base. There was recognition that reducing toxicity after drug administration was beneficial. With the lack of a body of support, professional GPs may feel that the implementation of pharmacogenomics may not be optimal. Barriers to implementation identified under the main themes are not unique to General Practice (Saul et al. 2017; McGrath and Ghersi 2016), and are also barriers to mainstreaming genomic practice among staff affiliated with primary care such as practice nurses (Brennan 2015) and pharmacists (Clemerson et al. 2006; Haga et al. 2016). However, the opportunities in implementation includes personalising medicine, to reduce toxicity, to enhance the efficacy of drugs and to use genome guided therapy in the setting of polypharmacy (Swinglehurst and Fudge 2017).

The Chief Medical Officer’s (CMO) recently published report on genomics, entitled Generation Genomics (Genomics and Therapeutics 2017), has a genomics and therapeutics theme which identifies the potential for stratified medicine so that new drug targets are identified and exploited for therapeutics which in turn can affect drug dosing and drug safety.

The future (next 5–10 years) holds the promise of advancing the use of genomic information in mainstream medicine and prescribing. The benefits include reduced toxicity and better efficacy. This will require a concerted effort with collaborative working between primary care, NHS England, Heath Education England, the UK Pharmacogenetics Stratified Medicines Network, NHS Digital and Clinical Genetics and others including the Royal Pharmaceutical Society to facilitate adoption.

The findings in this study cover broad generic issues that could equally be considered and extrapolated to other health care systems in other countries. We envisage that further studies around implementation that analyse each of the themed issues found in this study could inform the use of pharmacogenomics testing into clinical medicine that could lead to greater efficacy and reduced adverse drug events.

## Appendix. Semi-structured interviews

### Developing a Road Map for the implementation of Pharmacogenomics into UK General Practice: barriers, challenges and opportunities

- What do you understand about the term pharmacogenomics? Do you think it is a term that would be easily understand by most GPs?
- How do you think pharmacogenomics could help or hinder the GP in day to day practice (efficacy, reducing toxicity, time commitment, workflows?)
- What do you think could be the challenges in the use of genomic data? (clinical utility, interpretation, understanding).
- Are there any tests based on genomic information that is or could be helpful to practice?
- What are the necessary steps in terms of the use of genomic information in GP prescribing and treatment optimisation? (informatics, cost, timely etc.).
- What are the potential barriers to such implementation (patient factors?)
- What recommendations would you make based on the informatics needs of practices and practitioners.
- What will be necessary and need to be provided by the primary care IT system providers?
- Are there any education or training needs?
- Is there anything else you feel is relevant to the discussion?

Thank you.
